# Extraction, Enrichment, and LC-MS*^n^*-Based Characterization of Phlorotannins and Related Phenolics from the Brown Seaweed, *Ascophyllum nodosum*

**DOI:** 10.3390/md18090448

**Published:** 2020-08-27

**Authors:** J. William Allwood, Huw Evans, Ceri Austin, Gordon J. McDougall

**Affiliations:** 1 Plant Biochemistry and Food Quality Group, Environmental and Biochemical Sciences Department, The James Hutton Institute, Dundee DD2 5DA, UK; will.allwood@hutton.ac.uk (J.W.A.); Ceri.Austin@hutton.ac.uk (C.A.); 2 Byotrol Ltd., Thornton Science Park, Chester CH2 4NU, UK; hevans@byotrol.com

**Keywords:** phlorotannins, *Ascophyllum*, seaweed, health benefits, isomers, LC-MS*^n^*, diversity, phenolics

## Abstract

Phenolic components from the edible brown seaweed, *Ascophyllum nodosum*, have been associated with considerable antioxidant activity but also bioactivities related to human health. This study aims to select and identify the main phlorotannin components from this seaweed which have been previously associated with potential health benefits. Methods to enrich phenolic components then further select phlorotannin components from ethanolic extracts of *Ascophyllum nodosum* were applied. The composition and phenolic diversity of these extracts were defined using data dependent liquid chromatography mass spectroscopic (LC-MS*^n^*) techniques. A series of phlorotannin oligomers with apparent degree of polymerization (DP) from 10 to 31 were enriched by solid phase extraction and could be selected by fractionation on Sephadex LH-20. Evidence was also obtained for the presence of dibenzodioxin linked phlorotannins as well as sulphated phlorotannins and phenolic acids. As well as diversity in molecular size, there was evidence for potential isomers at each DP. MS^2^ fragmentation analyses strongly suggested that the phlorotannins contained ether linked phloroglucinol units and were most likely fucophlorethols and MS^3^ data suggested that the isomers may result from branching within the chain. Therefore, application of these LC-MS*^n^* techniques provided further information on the structural diversity of the phlorotannins from *Ascophyllum*, which could be correlated against their reported bioactivities and could be further applied to phlorotannins from different seaweed species.

## 1. Introduction

Phlorotannins are dehydro-polymers of phloroglucinol units particularly associated with brown seaweeds (Phaeophyceae) [1,2,3]. They appear to play a defensive role in the seaweeds, protecting against herbivory [4,5,6] and UV-B radiation e.g., [7]. Their levels vary with season, developmental stages and abiotic stresses [8,9,10,11] and it has been suggested that there is balance between a structural role in the algal cell wall and these protective roles [12]. They exist in structurally different forms depending on how the phloroglucinol units are interlinked [2]. If the phloroglucinol units are only linked by phenyl -C-C bonds, they are termed fucols; if they are only linked by -C-O-C- aryl ether bonds, they are termed phlorethols and if both linkages are present they are termed fucaphlorethols. Some phlorotannins also have dibenzodioxin linkages and these are generally termed as eckols. The type of inter-linkages of phlorotannins vary notably between species as is suggested by their names, *Fucus* species are rich in fucols and fucophlorethols and *Ecklonia* species notably contain eckols. Molecular size or the degree of polymerization of phlorotannins also varies greatly between species and may also be affected by biotic and abiotic stresses [2].

The phlorotannins have considerable antioxidant activity e.g., [13,14,15] and have been mooted as food-grade additives to prevent spoilage [16]. They have also been associated with specific health beneficial effects in related to disease states such as inflammation [17], cancers [18], diabetes [19] and hyperlipidemia associated with cardiovascular issues [20], which may be independent of their antioxidant activity. They have also been suggested to have valuable antimicrobial effects [21], with some efficacy against viruses e.g., [22]. The link between phlorotannin structure and activity has not particularly been well defined as many studies use extracts with varying extents of enrichment.

Their potential bioactivities are particularly relevant as brown seaweeds are generally edible and have formed part of the food culture of humans across the world, but notably in the Far East [23]. The edible brown seaweed *Ascophyllum nodosum* is common around the coasts of the UK and reaches high abundance around Scotland. Our previous work indicated that phlorotannins from *Ascophyllum* can inhibit digestive enzymes and thereby could modulate glycemic responses or reduce calorie intake from fats [18,24,25]. In this paper, we report on the enrichment and fractionation of phlorotannins and related phenolics from *Ascophyllum nodosum* and apply a series of liquid chromatography mass spectrometric (LC-MS*^n^*) methods to define their structural diversity.

## 2. Results and Discussion

### 2.1. Fractionation of Phenolic Material Using Solid Phase Extraction (SPE) and Sephadex LH-20

The total phenol content (TPC) of the *Ascophyllum* extract was mainly retained in the SPE-bound fraction with an overall recovery of ~90% of applied material in the fractionation (see Supplementary Data; Figure S1). The reasonably high TPC in the unbound material may be due to non-phenolic material that cross-reacts with the non-specific Folin reagent [26]. In the Sephadex LH-20 fractionation, the majority of the phenolic material was recovered in the first 80% acetone fraction and >80% of the total in the fractions released by acetone. The requirement for higher concentrations of acetone for release of bound material has been noted before [24]. Once again, the TPC in the unbound material may be due to non-phenolic material that cross-reacts with the non-specific Folin reagent.

### 2.2. Liquid Chromatography Mass Spectrometric (LC-MS^n^) Analysis

**Figure 1:**
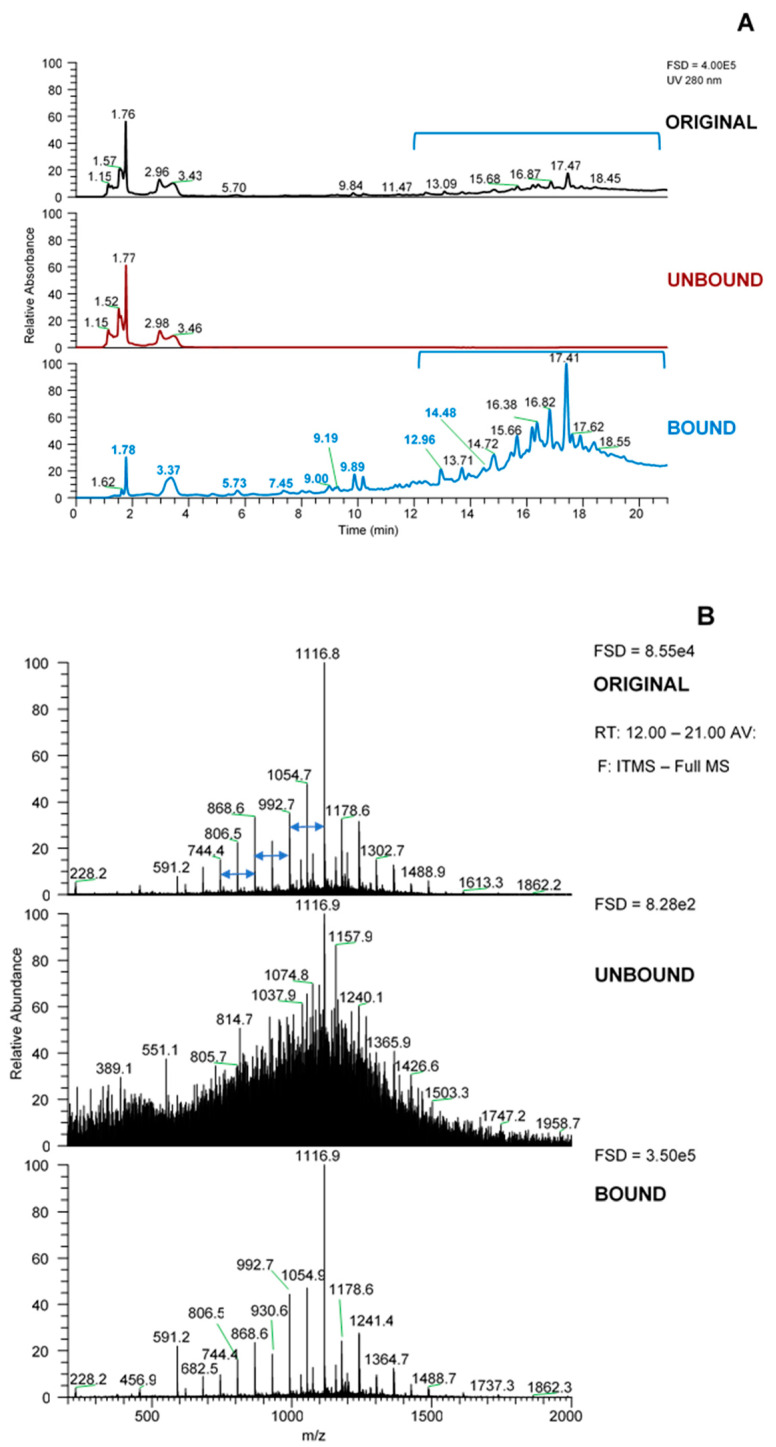
Profiles of the fractions from the SPE procedure. (**A**) UV profiles, (**B**) MS spectra from 12–21 min for each sample. Blue bracket in (**A**) represents area for MS spectra in (**B**). Arrows show 124 amu differences between peaks. FSD = full scale deflection.

**Figure 2:**
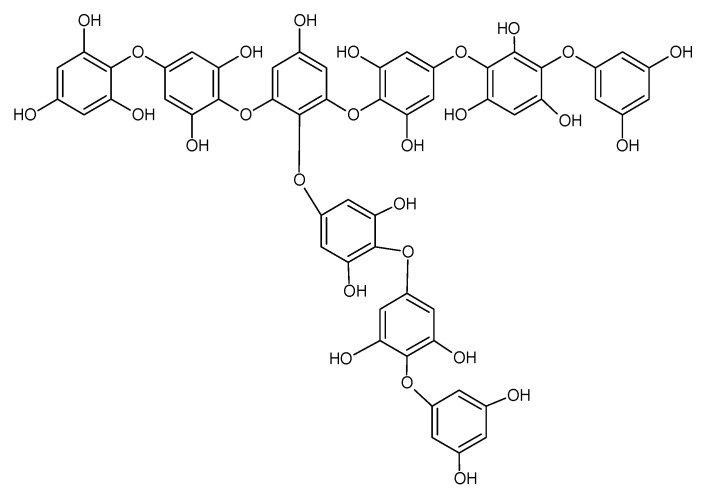
Possible nonaphorethol structure.

Using the LC-MS data acquired on the LCQ Fleet MS system, the SPE bound sample was enriched in later eluting UV-absorbing peaks (Figure 1A) whereas the unbound fraction was essentially devoid of these peaks. The MS spectrum across the retention time of the UV peaks from 12–21 min (Figure 1B) gave a set of *m*/*z* signals in negative mode which were characteristic of phlorotannins. These could be thought of as two series of *m*/*z* values that differ by 124 amu, an extension unit mass equivalent to phloroglucinol minus 2 H atoms, which have been noted previously in our laboratory [18,24,25], or indeed as a single series that differ by 62 amu (124 divided by 2). If the *m*/*z* values from 621, 745, 869, 993, 1117, 1241, 1365 and 1489 were single charged then they could arise from successive phloroglucinol additions to a phloroglucinol dimer with *m*/*z* [M − H]- of 249 (such as diphlorethol) and working on this basis, the signals at *m*/*z* 621 and 745 could be pentaphloroethol and hexaphloroethol structures and the major signal noted at *m*/*z* 1117 could be a nonaphloroethol derivative (see Figure 2) [2]. However, if we assume singly charged ions then the possible nature of the *m*/*z* series at 683, 807, 931, 1055, 1179, 1303 etc. is not apparent unless all the signals were from double charged species. However, it was not possible to confirm the charge status of the ions as the resolution of the Fleet LCQ MS cannot discern this in normal mode function.

**Figure 3:**
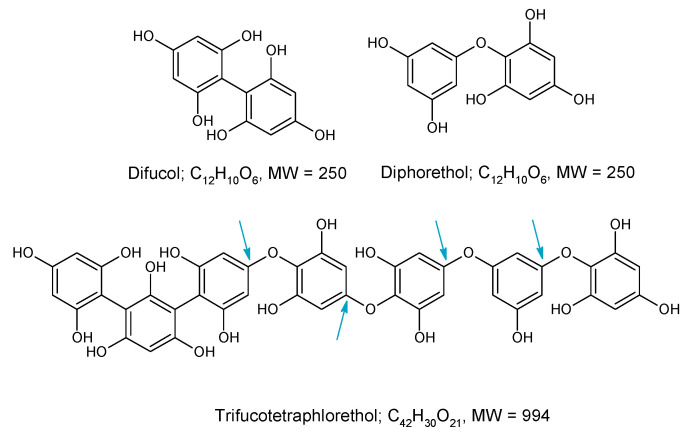
Structures of difucol, diphlorethol and trifucotetraphlorethol. These structures are discussed in the text. Fragmentation at the positions noted with blue arrows would produce THB containing fragments with neutral losses of 142 amu = THB, 266 amu = PG-THB, 390 amu = 2PG-THB and 514 amu = 3PG-THB respectively from right to left.

The phlorotannin series outlined above is based on a phlorethol structure, oligomers of phloroglucinol attached through aryl ether (-C-O-C) bonds but the addition of phloroglucinol units (124 amu) to achieve this series could also be achieved through phenyl (-C-C-) linkages (see Figure 3). In fact, this series of *m*/*z* values could arise from phloroglucinol units linked by all phenyl linkages (called fucols), all aryl ether linkages (called phlorethols) or a mixture of both (called fucophlorethols) [2,15].

**Figure 4:**
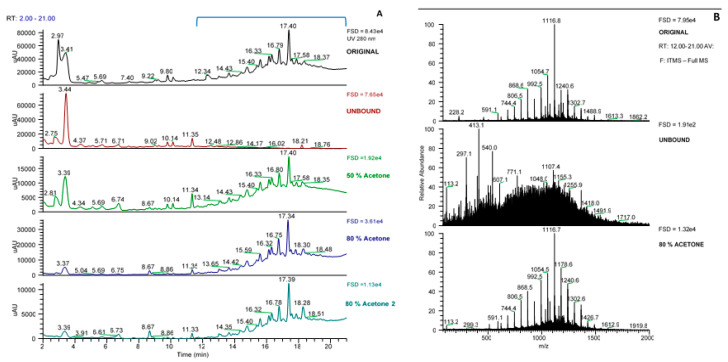
Profiles of the fractions from the Sephadex LH-20 procedure. (**A**) UV profiles, (**B**) MS spectra from 12–21 min for each sample. FSD = full scale deflection.

It was notable that these “phlorotannin” MS signals were approximately 4-fold enriched in the bound fraction obtained after SPE (see FSD in Figure 1B). Also, the bound acetone fractions from the Sephadex LH-20 separation also showed this enrichment in UV-absorbing peaks between 12 and 21 min (Figure 4A). However, in this case, the enrichment was less apparent as the phlorotannins were spread over the three acetone fractions and there was a dilution factor inherent in the procedure. However, these UV peaks contain the same set of *m*/*z* signals characteristic of phlorotannins (Figure 4B) and these are sufficient at this stage to follow the enrichment of these phlorotannin species.

### 2.3. Differences between Positve and Negative Mode MS Data

**Figure 5:**
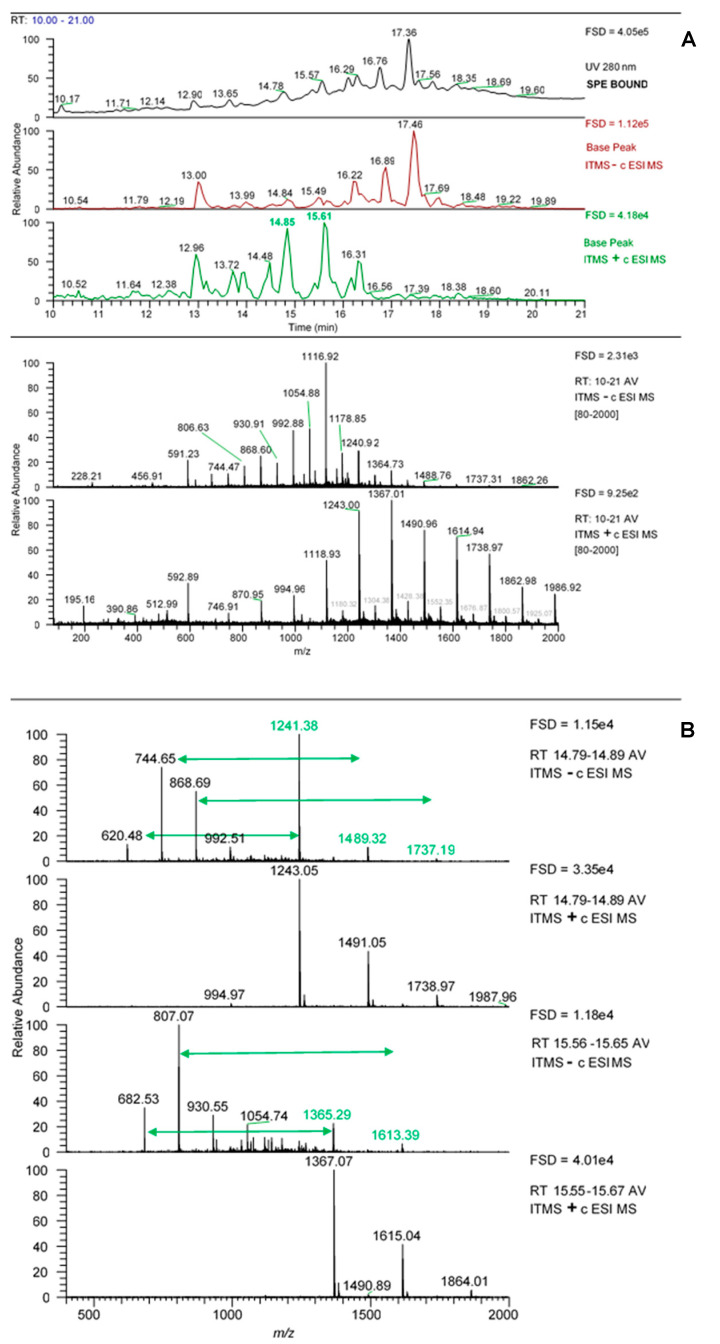
(**A**) MS properties of phlorotannin peaks in negative and positive mode. The top panel shows the UV spectra of the SPE bound sample from 10–21 min, the next shows the MS spectra in negative mode from 10–21 min and the third panel shows the MS spectra in positive mode from 10–21 min. The fourth panel shows the MS spectrum over 10–21 min in negative mode and the last panel shows the same in positive mode. FSD = full scale deflection. The peaks denoted in bold green were examined further (Figure 5B). (**B**). MS spectra of selected phlorotannin peaks in negative and positive mode. Examples of negative and positive MS spectra of specific peaks labelled in bold in Figure 5A. Green arrows denote *m*/*z* signals discussed in the text.

Using the Fleet LCQ MS system, the MS profiles of the phlorotannin peaks in the SPE bound sample were different in negative and positive mode (Figure 5A, panels A–C). In general, intensities were lower in positive mode but there were qualitative differences in the ionization of different peaks. For example, the major peak in negative mode is at 17.46 min and the MS profile largely matches the UV profile (compare panel A and B). In positive mode, it is notable that the later eluting major UV peaks (e.g., RT = 16.8 and 17.4) have lower relative MS intensity then the negative mode data. The negative mode MS data across RT 10–21 min showed the series of major peaks that differ by 62 amu (panel D). The positive mode MS data over the same retention range showed a series beginning at *m*/*z* 747 that increased by 124 amu through 871, 995, 1119, etc. However, there was also a minor series of *m*/*z* signals that began at 1180 (in grey) and also increased by 124 amu. When the MS data under specific phlorotannin peaks was examined, the situation became clearer. The spectra under the MS peaks shown in bold in Figure 5A are shown in negative mode then positive mode in Figure 5B. The MS peak at ~14.8 in positive mode had three main signals at *m*/*z* 1243, 1491 and 1739. In negative mode, the same peak gave the corresponding *m*/*z* [M − H]^−^ signals at 1241, 1489 and 1737 but also had strong signals at 620, 746 and 868 which were effectively half the values of the other signals. The other major peak at RT 15.6 showed a similar picture. Therefore, in both examples, the negative mode MS spectra contained *m*/*z* signals that are effectively half the *m*/*z* value of the positive mode signals. This disparity can be explained if the phlorotannins ionise mainly as singly charged ions in positive mode (i.e., as *m*/*z* [M + H]^+^ ions) but they have a propensity to ionise as doubly charged ions (i.e., as [M − 2H]^2−^ ions) in negative mode. In fact, this can be neatly illustrated by the co-chromatography of peaks with base peaks at *m*/*z* [M − H]^2−^ values against [M + H]^+^ ions in the SPE bound sample (see example in Figure S2).

As noted above, operating in normal full scan mode, the Fleet MS system cannot discriminate between single and double charged ions. Therefore, we re-examined the samples using the FT-MS detector of an LTQ Orbitrap XL system that has sufficient resolution to differentiate 0.5 amu isotope spacing, as observed in double charged ions.

### 2.4. Re-Examination of Data Using the LTQ-Orbitrap XL FT-MS System

**Figure 6:**
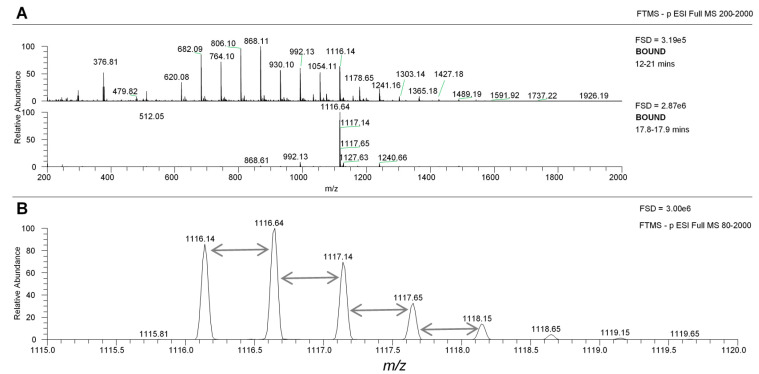
The phlorotannin peaks yield doubly charged MS signals in negative mode. The top panel (**A**) shows the MS spectra across 12–21 min of the SPE bound sample and the second panel shows the MS spectra of the main UV peak. The third panel (**B**) shows the zoom spectra across the *m*/*z* 1116 peak.

When analyzed on the LTQ-Orbitrap XL system in ESI negative mode, the major peak at RT 17.8 showed revealed ions that differed by 0.5 amu, indicating that they are double charged ions (i.e., *m*/*z* [M − 2H]^2−^, Figure 6). As a double charged ion, the true mass would be twice as high at 2234, and this suggests that the main UV peak at RT 17.8 actually contained an oligomer of 18 phloroglucinol (PG) units rather than the nonaphloroethol suggested before. In fact, all of the *m*/*z* values in the negative mode series from *m*/*z* 621 upwards were doubly double charged which indicates that they are all double the MW than first suggested by the original LCQ-Fleet MS data. It also confirms that the original observed peak spacing of 62 amu observed upon the LCQ-Fleet MS system, when accounting for the ions being double charged, in fact represents a single series of ions with peak spacing of 124 amu, corresponding to phloroglucinol minus 2 H atoms. Peaks for all the ion species with apparent double charged [M − 2H]^2−^ ions from *m*/*z* 621 through to *m*/*z* 1429 were identified in the MS profiles of the SPE bound samples (see Figure S3). At least two main peaks were apparent for each *m*/*z* species which suggests the presence of isomers of the different oligomers of each DP.

**Figure 7:**
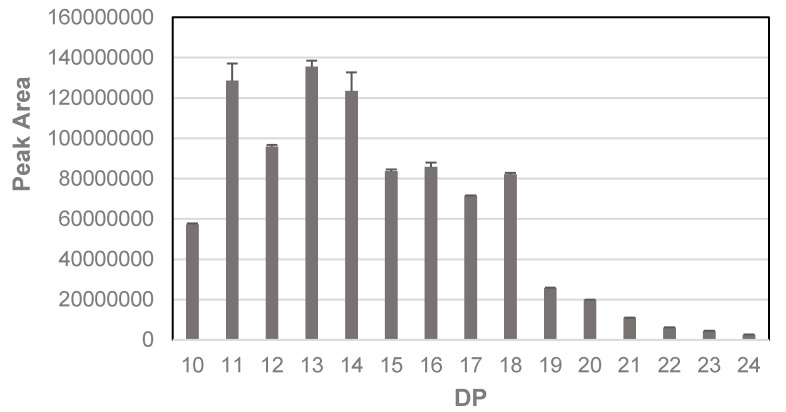
Relative abundance of phlorotannin oligomers in the SPE bound fraction.

For quantification, we summed the areas for the major putative isomer peaks but ignored other small peaks which may be due to in-source fragmentation within the MS spectra of other oligomers. The relative abundance of these putative phlorotannin oligomers from DP 10 to 24 are shown in Figure 7. Plotted against DP, the oligomers of DP 11–18 were the most abundant with a drop-off at DP > 18. In fact, although some peaks areas for *m*/*z* values for phlorotannin oligomers >24 DP could be discerned, they were not significantly above baseline values. However, there were indications that phlorotannins of larger DP were present in the region between RT 19–21 min where triple charged *m*/*z* signals could be discerned (see Supplementary Data, Figure S4) that suggested the presence of oligomers of up to DP 31 (e.g., a triple charged ion at 1281.42 yields an estimated MW of 3846). However, these were in much lower abundance and did not yield MS^2^ data. Previously, the presence of triple charged phlorotannin species has been indicated [27] and the distribution and range of DP noted in our work fits in the range noted by this group.

It is important to note that as well as having more intense signals, the negative mode data with its double and triple charged ions actually allowed the detection of molecular species with MWs > 2000 amu that would not have been detected in positive mode. The positive mode analysis would have been limited to the detection and quantification of phlorotannins with DP = 16 PG units (*m*/*z* [M + H]^+^ = 1987). This also explains why the later eluting phlorotannins noted above had such poor MS spectra in positive mode as their signals were effectively outside the detectable MS range of 100–2000 *m*/*z*. Indeed, Tierney et al. [28] reported that phlorotannins from *Ascophyllum* had a lower DP range, with a maximum of 16 PG units. Although they applied a dialysis step which may have altered the MW range, they crucially used an MS detector which limited detection at DP 16 (i.e., a *m*/*z* value = 1987). However, a previous paper by this group [29] detected phlorotannins of up to DP 20 in low molecular weight extracts of *Ascophyllum nodosum* by using direct infusion of SPE-purified extracts into a Q-Tof Premier mass spectrometer with detector mass range of *m*/*z* 100 to *m*/*z* 3000.

The MS data obtained on the LTQ-Orbitrap XL system also provided evidence for the presence of other phlorotannin components. For example, there was a peak at RT = 12.96 (see Figure 1B, bound sample, bold blue peaks), which preceded the cluster of phlorotannin oligomers. This gave *m*/*z* [M − H]^−^ = 591.2 with fragmentation yielding MS^2^ fragments of 511 & 385 and an accurate mass of 591.0075 derived a molecular formula of C_24_H_15_O_16_S at < 1 ppm error (Table 1). The neutral loss of 80 amu can be assigned to loss of a sulphate group (SO_3_) to a compound with formula *m*/*z* [M − H]^−^ of C_24_H_15_O_13_, which matches with diphlorethohydroxycarmalol (Pub Chem 16075395), a phlorotannin component noted in the brown seaweed, *Ishige okamurae* [30]. The presence of this diphlorethohydroxycarmalol derivative was suggested but not confirmed in our previous studies of *Ascophyllum* [23,24]. In fact, a peak attributable to diphlorethohydroxycarmalol itself (RT = 14.48; *m*/*z* [M − H]^−^ = 511.0506, predicted formula of C_24_H_15_O_13_ and sole MS^2^ fragment at 385; Table 1). was also present later in the separation. There was also evidence for the presence of a component with *m*/*z* = 246.9914, MS^2^ = 203, 121, with a predicted formula C_12_H_7_O_6_, that matched with the dimer, dibenzodioxin-1,3,6,8-tetraol, which has been reported previously in *Fucus* species e.g., [27]. These three phlorotannin components contain a dibenzodioxin-ring structure which has not been noted previously in *Ascophyllum*. There were also other components which appeared to be sulphated phenolic acids and another whose MS and MS^2^ properties matched with a DOPA-sulphate-like component. However, confirmation of the identity of these components requires further isolation and characterization by techniques such as 2D NMR. It was notable that all these components were enriched by the SPE procedure but were greatly reduced by the selection of the phlorotannin oligomers on Sephadex LH-20.

### 2.5. Structural Information from MS^2^ Fragmentation Data

Different collision energies were assessed to maximize the yield of MS^2^ fragments and to increase the likelihood of providing useful structural data for the phlorotannin structures. The NCE of 45% applied in our standard MS^2^ method often only provided weak fragmentation spectra and overall, better fragmentation required an NCE of 65% which was adopted for further analyses. The higher energies required for effective fragmentation probably reflect the stable nature of the phlorotannin oligomers. Fragmentation data was available for all peaks corresponding to phlorotannin oligomers up to DP = 23 at *m*/*z* [M − 2H]^2−^ = 1427, which did not provide MS^2^ data as it was below the MS^n^ minimum trigger intensity (Table 2). The fragmentation data for the phlorotannins was characterized by several common factors. Firstly, as all the target *m*/*z* values were double charged, there were fragments greater than the *m*/*z* [M − 2H]^2−^ value. Secondly, often the major fragment resulted from the *m*/*z* [M − 2H]^2−^ value minus H_2_O (neutral loss of 18 amu). Overall, the MS^2^ data gave fragments and neutral losses previously noted in reports of phlorotannin structures [31,32,33,34,35,36]. Notably, the neutral losses obtained through fragmentation showed patterns between the different phlorotannin structures with repeated neutral losses being observed (Table 2), which could largely be assigned to the loss of a fixed numbers of phloroglucinol (PG) groups or PG groups + H_2_O. It was also notable that major fragments noted for different phlorotannin components that differed by 124 amu in full scan MS, also generated fragment ions that differed by 124 amu, suggesting that a consistent fragmentation mechanism was occurring. Other undefined fragmentations in Table 1 could arise through ring fission events within PG units. It was also notable that major fragments noted for different phlorotannin components that differed by 124 amu also differed by 124 amu, which suggests that a consistent fragmentation mechanism was occurring. Other undefined fragmentations in Table 2 could arise through ring fission events within PG units [28,29,34].

Another important finding was the commonality of fragmentations that could be explained by the presence of a tetrahydroxylbenzene structure in the neutral loss (also called O-phloroglucinol moieties previously e.g., [34]. For example, a neutral loss of 514 amu was noted in the MS^2^ spectra of many of the phlorotannin species (Table 2) and this could arise if the phlorotannin molecule fragmented at an ether bond and formed a phloroglucinol with an extra hydroxyl group linked to a trimer of PG units (i.e., 3PG + THB = 374 + 140; see Figure 3). In fact, this neutral loss (514 amu) is the same as the molecular weight of tetrafuhalol, a trimer of phloroglucinol units linked to a tetrahydroxybenzene unit [2], an example of another type of phlorotannin found in other brown seaweeds. Indeed, neutral losses consistent with one PG unit attached to one THB unit (i.e., 266 amu) up to 16 PG units attached to a THB group (i.e., 2126 amu) were indicated in the MS^2^ fragments of the phlorotannins (Table 2). The only fragments missing from this series were those which represent 9, 10 and 12 PG units linked to a THB group and a THB group itself. THB-containing neutral losses were present in the MS^2^ of every phlorotannin at each DP examined (Table 2) and since they can only occur at -C-O-C- ether linkages [31,32,33,34], this strongly suggests that the phlorotannins in *Ascophyllum* are fucophlorethol-type components. Certainly, evidence of at least one aryl ether bond in the oligomers means that they cannot be fucol oligomers, which are composed of only phenyl linked PG units. The possibility that cleavage of C-C phenyl bonds requires more energy than cleavage of C-O-C ether bonds has been discussed previously [2,34] and this may explain the preponderance of cleavages at ether bonds.

### 2.6. Structural Information from MS^3^ Fragmentation Data

**Figure 8:**
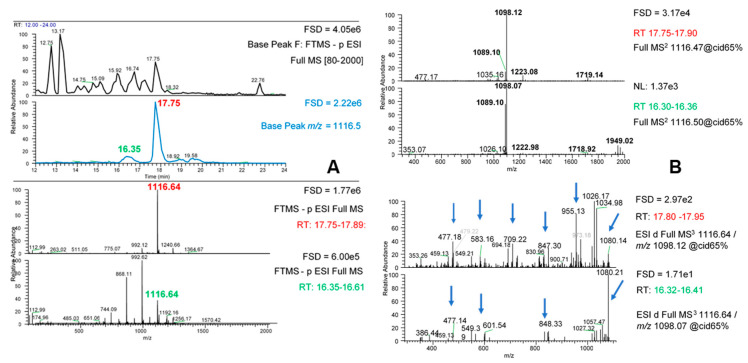
MS^2^ and MS^3^ data of major phlorotannin isomers at *m*/*z* 1116.6. (**A**) The top panel shows the MS profile between RT = 21–21 min, the second panel shows the profile for base peak at *m*/*z* 1116.6 and the bottom two panels show the MS spectra at the two peaks at 17.75 min and 16.35 min respectively. (**B**) The top panel shows the MS^3^ fragments from the main MS^2^ peak (*m*/*z* 1098) derived from the major *m*/*z* 1116.6 peak at RT = 17.75. The bottom panel shows the MS^3^ fragments from the main MS^2^ peak (*m*/*z* 1098) derived from the minor *m*/*z* 1116.6 peak at RT = 16.35.

Further information was sought by acquiring MS^3^ fragmentation data from the MS^2^ fragments for each phlorotannin species. Once again, application of 45% NCE as defined in our original method did not provide strong and consistent compound fragmentation, therefore the NCE was increased to 65% for each MS^2^ target. Using the [M − 2H]^2−^ ion at *m*/*z* 1116.6 as an example (Figure 8), there was a major and a minor peak at *m*/*z* 1116.6 at RT 17.7 and RT 16.3 respectively which may represent isomers (Figure 8A). Indeed, the presence of 2 or 3 isomers for each phlorotannin signal was noted previously (Figure S3) and the different chromatographic behaviour of these apparent isomers suggests structural differences. The different peaks gave different MS^2^ patterns. The more abundant isomer gave *m*/*z* 1098 as the predominant fragment whereas the less abundant isomer gave mainly *m*/*z* 1098 and 1089 but with other MS^2^ products in more equal amounts. Once again, both isomers gave MS^2^ products greater than the original target *m*/*z* value (e.g., *m*/*z* values of 1223, 1719 and 1949) due to their double charged nature. Similarly, the major and minor isomers of the other phlorotannin peaks gave different MS^2^ patterns (results not shown).

The MS^3^ fragments obtained by fragmentation of the dominant MS^2^ signal at *m*/*z* 1098 for the two *m*/*z* 1117 isomers are shown in Figure 8B. As expected, many of these MS^3^ fragments were also found as minor fragments in the MS^2^ spectra. It was noticeable that the MS^3^ products of the *m*/*z* 1098 MS^2^ fragment for the different isomers were also different. The more minor isomer at RT 16.3 gave a simpler MS^3^ pattern than the isomer at RT 17.8. The major isomer gave MS^3^ products for *m*/*z* 1098 which were spread across apparent losses of 1, 2, 3, 4 and 5 PG units whereas the MS^3^ products from the *m*/*z* 1098 MS^2^ fragment from the minor isomer gave a major neutral loss of H_2_O to *m*/*z* 1080 with smaller amounts of fragments resulting from losses of apparent 2, 4 and 5 PG units (see arrows, Figure 8B). The different pattern of losses at the MS^3^ level suggest that these apparent phlorotannin isomers differ in their inter-linkages. For example, the major isomer seems to break into smaller fragments which could mean a more branched structure, perhaps with more ether bonds. However, confirmation of such differences would require purification of the isomers and further studies, perhaps using 2D-NMR approaches.

MS^3^ fragmentation data from the other notable MS^2^ fragments from the *m*/*z* 1116.6 isomers could yield further information, but due to their lower ion intensities, MS^3^ fragmentation data was not obtainable. The MS^2^ product of *m*/*z* 1719 gave a single product ion at *m*/*z* 892 which suggests a clean fragmentation with a neutral loss of 827. However, the MS^3^ DDA of the MS^2^ product at *m*/*z* 1223 gave multiple MS^3^ ions. Given that the intensities of these MS^2^ products varied in abundance between the two isomers, this further suggests differences in structure. Overall, the MS^2^ evidence (Table 2) strongly suggested that the phlorotannin oligomers were fucophlorethols as they contained aryl ether linkages [2]. Due to the three-way symmetry of the phloroglucinol molecule, -C-C- phenyl linkages are always effectively meta-orientated [15]. However, PG units attached through aryl ether bridges can occur at hydroxyls at ortho, meta or para positions on the rings so the possibilities for different isomeric structures are large especially as the DP increases. For example, extending from a diphorethol structure to a triphlorethol structure can produce structurally discernible triphlorethol A and B isomers [2] depending on the positioning of the “new” aryl ether bond. Also, branching can also occur within fucol type phlorotannins composed of only phenyl linkages if one of the PG units has 3 rather than 2 linkages to other PGs [2,15]. Indeed, other studies have reported multiple apparent isomers of phlorotannin oligomers from *Ascophyllum* [28,29,31] and other brown seaweeds [37]. In fact, the presence of only two (perhaps three maximum) major isomers as noted in this paper seems unusual compared to the 70 apparent isomers for a DP 10 oligomer reported in one study [29]. However, it is also possible that some of the isomers noted result from in-source fragments of larger phlorotannins, or from a greater number of different charge states being generated in ESI and higher MS scan ranges, which might be more apparent under the different MS conditions used in each study. Indeed, it is also possible that our chromatographic procedure did not separate all these different putative isomers, but the chromatographic conditions used similar C18 reverse phase conditions. It also seems particularly unlikely that our extraction procedure would have so drastically influenced isomer diversity.

## 3. Conclusions

The biosynthetic pathway by which brown seaweeds produce phloroglucinol via the acetate-malonate pathway, also known as the polyketide pathway, is understood, in a process which involves a specific polyketide synthase-type enzyme [37]. However, the mechanisms by which phloroglucinol groups are combined in such a controlled manner, especially to yield the diversity of phlorotannin structures found in different species [2], and the extent that the oligomers extend are less well understood. The LC-MS^n^ techniques used here have yielded useful information of the possible structural diversity of the phlorotannin oligomers in *Ascophyllum*, and further advances could be made, especially if ion-tree based fragmentation methods [38] could also be employed. However, the level of complexity for the major phlorotannin component (*m*/*z* [M − 2H]^2−^ = 1117; apparent MW 2234; possible fucaphlorethol of DP 18) highlighted in this paper illustrates the daunting extent of data interpretation required to make inferences of overall structure. Also, MS-based analyses are inherently limited by their mass range and cannot deal with the high molecular weight phlorotannin species known to be present in brown seaweeds [2,15]. However, the molecular range could be extended by using other MS systems with higher MW ranges such as MALDI-TOF, as described previously e.g., [32]. Further work could focus effort on the main phlorotannin oligomers as these are most likely to be those most associated with specific bioactivities noted for the phlorotannin samples. In addition, future work could use these techniques to examine phlorotannins from different species of brown seaweed and begin to correlate their structural diversity with potential biological effectiveness.

## 4. Materials and Methods

### 4.1. Materials and General Methods

Dried and milled *Ascophyllum nodosum* powder was obtained from Hebridean Seaweeds in 2017 and stored at −20 °C until use. The ASCO extract was prepared from this dried powder using a propriety hydro-ethanolic extraction procedure carried out by Byotrol plc. The extract was filtered then reduced in volume by rotary evaporation until it could be freeze dried to a powder. The freeze-dried extract was soluble at 10% (*w*/*v*) in ultra-pure water.

Total phenol content (TPC) was assessed using the Folin-Ciocalteu method [24]. The TPC of the original extract was 0.394 ± 0.18 g GAE/g DW. It should be noted that the Folin method is not totally specific and these values probably over-estimate the yield of phenolic components.

### 4.2. Solid Phase Extraction

The method used was developed and scaled up from that reported previously [18]. In brief, a SPE unit (Strata C18-E GIGA tube, 10 g capacity & 60 mL volume; Phenomenex Ltd., Macclesfield, UK) was washed with 2 × 50 mL volumes of acetonitrile (ACN) containing 0.1% formic acid (FA) then equilibrated with 3 × 50 mL of UPW containing 0.1% FA.

The FD extract was dissolved at 5% (*w*/*v*) in UPW containing 0.1% FA and applied to the SPE unit, the unbound fraction was recovered, then the SPE unit was washed with 2 × volumes of UPW + 0.1% (*v*/*v*) FA, collected as the wash fraction. The bound fraction was obtained by eluting the unit with 2 volumes of 80% ACN + 0.1% (*v*/*v*) FA. The unit was then re-equilibrated for further use by washing with UPW + 0.1% (*v*/*v*) FA. At this stage, it was noted that the eluted fraction was cloudy so this bound-RW (rewash) fraction was also collected. The fractions were tested for total phenol content. Aliquots of each fraction were completely dried by speed vacuum concentration for LC-MS*^n^* analysis.

### 4.3. Fractionation on Sephadex LH-20

A portion of the *Ascophyllum* extract was fractionated using Sephadex LH-20 applying a technique [23] well-known to select for phlorotannin-like components (https://www.users.miamioh.edu/hagermae/). In brief, a 25 mg/mL solution of the FD material in UPW was produced and then 5 mL was added to 5 mL ethanol and mixed well. This solution was added to 5 mL of a slurry of Sephadex LH-20 in 50% ethanol and mixed well for 10 min at room temperature. After centrifugation at 2500× *g* for 5 min at 5 °C, the unbound fraction was removed, and 5 mL of 50% ethanol added. The centrifugation procedure was repeated to give the wash fraction then similarly with 50% acetone and then two washes with 80% acetone to provide the bound fractions. The total phenol contents were measured as before, and aliquots of each fraction completely dried by centrifugal evaporation in a Speed Vac prior to LC-MS*^n^* analysis.

### 4.4. Liquid Chromatography-Mass Spectrometry (LC-MS^n^) Analysis

The samples were first analyzed on an LCQ Fleet Ion Trap mass spectrometer (Thermo Scientific Ltd., Hemel Hempstead, UK) attached to an HPLC system consisting of an Accela 600 quaternary pump and Accela photodiode array detector (PDAD) and autosampler. Spectra were collected in wavelength/absorbance mode 200–600 nm (1nm filter bandwidth and wavelength step, 1 s filter rise time, 10 Hz sample rate). Additionally, three UV channel set points were employed (A: 280 nm, B: 365 nm, C: 520 nm, 9 nm bandwidth, 10 Hz sample rate). Samples (20 μL injection volumes) were eluted on a Synergi Hydro C18 2.0 mm × 150 mm, 4 µm particle size column (Phenomenex Ltd., Macclesfield, UK) applying mobile phase A, HPLC grade water + 0.1% FA, and mobile phase B, HPLC grade Acetonitrile + 0.1% FA, at a flow-rate of 0.3 mL/min. The gradient was as follows: 0–2 min hold 2% B, 2–5 min 2–5% B, 5–25 min 5–45% B; 25–26 min 45–100% B, 26–29 min hold 100% B, 29–30 min 100–2% B, 30–35 min hold 2% B for HPLC equilibration. Mass spectra were collected with a primary full scan event (*m*/*z* 80–2000, profile mode) and a secondary data-dependent analysis (DDA) MS/MS scan (centroid mode) for the top three most intense ions. Helium was applied as a collision gas for collision-induced dissociation at a normalized collision energy (NCE) of 45%, a trapping window width of 2 (+/−1) *m*/*z* was applied, an activation time of 30 ms and activation Q of 0.25 were applied, only singly charged ions were selected for DDA, isotopic ions were excluded. The Automatic Gain Control was set to 1 × 104, scan speed to 0.1 s, the following settings were applied to ESI: Spray voltage −3.5 kV (ESI−) and +4.0 kV (ESI+); Sheath gas 60; Auxiliary gas 30; Capillary voltage at −35 V (ESI−) +35 V (ESI+); Tube lens voltage −100 V (ESI−) and +100 V (ESI+); Capillary temperature 280 °C; ESI probe temperature 100 °C.

Selected samples were separated using the same chromatographic conditions and PDA set points, but with a Thermo Dionex U3000 UHPLC-PDA (Thermo Fisher Scientific UK), coupled to a Thermo LTQ-Orbitrap XL mass spectrometry system capable of full scan accurate mass FT-MS (30,000 FWHM resolution defined at *m*/*z* 400), as well as DDA at MS^2^ and MS^3^ levels. The Orbitrap XL applied identical settings as the LCQ-Fleet, but with a scan speed of 0.1 s and 0.4 s and AGC of 1 × 10^5^ and 5 × 10^5^ for the LTQ-IT and FT-MS respectively. For selected samples, in addition to full scan accurate mass FT-MS and LTQ-IT MS^2^, LTQ-IT MS^3^ data were also collected in DDA mode for the top three most intense ions detected in each MS^2^ scan. MS^2^ and MS^3^ data were collected at collision energies of 45% and 65% NCE.

## Figures and Tables

**Table 1:** MS properties of phenolic components from *Ascophyllum nodosum.*

RT	*m*/*z* [M − H]^−^	MS^2^	Exact Mass Formulae as [M − H]^−^	Putative Identity
1.78	191.0195	173, **111** *	C_6_H_7_O_7_	Citric acid
3.37	276.0184	259, 231, **215**, **196**, 179, 150, 135	C_9_H_10_O_7_NS	Dihydroxyphenylalanine (DOPA)-sulphate (PC 178810)
5.73	246.9914	203, **121**	C_12_H_7_O_6_	Dibenzodioxin-1,3,6,8-tetraol (PC 14309078)
7.45	277.0924	**185**, 167, 141, 97	C_11_H_17_O_8_	Unknown
9.08	318.0284	300, 276, **238**, 192	C_11_H_12_NSO_8_	Unknown
8.93	216.9809	173, **137**, 97	C_7_H_5_O_6_S	Hydroxybenzoic acid sulphate
9.89	230.9967	187, **151**	C_8_H_7_O_6_S	Phenolic acid sulphate
12.96	591.0075	511, **385**	C_24_H_15_O_16_S	Diphlorethohydroxycarmalol sulphate
14.48	511.0506	**385**	C_24_H_15_O_13_	Diphlorethohydroxycarmalol (PC 16075395)

RT = retention time. All formulae were derived at <2 ppm error. * major fragments are in bold. PC = Pub Chem reference number.

**Table 2:** MS^2^ fragmentation properties for the phlorotannins of DP 10–23.

Phlorotannin			MS Properties				Phlorotannin			MS Properties			
***m*** **/*z*** **[M − 2H]^2−^**	**Calc *m*/*z***	**DP**	**MS^2^**	**NL**	**ΔM − 2H^2−^**	**Neutral** **Loss**	***m*** **/*z*** **[M − 2H]^2−^**	**Calc. *m*/*z***	**DP**	**MS^2^**	**NL**	**ΔM − 2H^2−^**	**Neutral Loss**
**621**	1241	10	1097	144		PG+H_2_O	**683**	1365	11	**1117**	248		2PG *
			993	248		2PG*				975	390		2PG-THB
			831	410		UK				851	514		3PG-THB
			603	638	18	4PG-THB				**664**	**701**	18	?
			**495**	**746**	125	6PG				610	755	72	?
			477	764	144	6PG+H_2_O				477	888	205	7PG+H_2_O
			247	994	373	8PG				371	994	311	8PG
			229	1012	391	8PG+H_2_O				355	1010	327	7PG-THB
										**229**	**1136**	453	9PG+H_2_O
**745**	1489	12	1345	144		PG+H_2_O							
			**1223**	**266**		PG-THB	**807**	1613	13	1469	144		PG+H_2_O
			975	514		3PG-THB				1223	390		2PG-THB
			**727**	**762**	18	5PG-THB				955	658		?
			672	817	72	?				**788**	**825**	18	?
			477	1012	267	8PG+H_2_O				725	888	81	7PG+H_2_O
			229	1260	515	10PG+H_2_O				477	1136	329	9PG+H_2_O
										353	1260	453	10PG+H_2_O
**869**	1737	14	1471	266		PG-THB							
			1223	514		3PG-THB	**931**	1861	15	1700	161		?
			975	762		5PG-THB				1595	266		PG-THB
			**851**	**887**	18	7PG+H_2_O *				1347	514		3PG-THB
			787	950	81	?				1099	762		5PG-THB
			477	1260	391	10PG+H_2_O				**912**	**949**	18	?
			353	1384	515	11PG+H_2_O				849	1012	81	8PG+H_2_O
										477	1384	453	11PG+H_2_O
**993**	1985	16	1595	390		2PG-THB				355	1506	575	11PG-THB
			1223	762		5PG-THB							
			**974**	**1011**	18	8PG+H_2_O *	**1055**	2109	17	1929	180		?
			911	1074	81	?				1719	390		2PG-THB
			848	1137	144	9PG+H_2_O				1469	640		5PG+H_2_O
			477	1508	515	12PG+H_2_O				1223	886		6PG-THB
			353	1632	639	13PG+H_2_O				**1036**	**1073**	18	?
										973	1136	81	9PG+H_2_O
**1117**	2233	18	1985	248		2PG *				831	1278	223	10PG+ 2H_2_O
			1719	514		3PG-THB				477	1632	577	13PG+H_2_O
			1451	782		?				353	1756	701	14PG+H_2_O
			1223	1010		7PG-THB							
			**1098**	**1135**	18	9PG+H_2_O*	**1179**	2357	19	1825	532		?
			1089	1144	28	?				1595	762		6PG+H_2_O
			1035	1198	81	?				1223	1134		8PG-THB
			709	1524	407	?				**1160**	**1197**		?
			477	1756	639	14PG+H_2_O				**1151**	**1206**	27	?
			337	1896	779	?				973	1384	205	11PG+H_2_O
										601	1756	577	14PG+H_2_O
**1241**	2481	20	1967	514		3PG-THB				479	1878	699	14PG-THB
			1719	762		5PG-THB				355	2002	823	15PG-THB
			1489	992		8PG							
			**1222**	**1259**	18	10PG+H_2_O*	**1303**	2605	21	1805	800		?
			1159	1322	81	?				1595	1010		7PG-THB
			955	1526	285	?				**1284**	**1321**	18	?
			727	1754	513	13PG-THB				1275	1330	27	?
			477	2004	763	16PG+H_2_O				**1221**	**1384**	81	11PG+H_2_O
			355	2126	885	16PG-THB				955	1650	347	?
										831	1774	471	14PG+2 H_2_O
**1365**	2729	22	1967	762		5PG-THB				479	2126	823	16PG-THB
			1719	1010		7PG-THB				365	2240	937	?
			1451	1278		?							
			**1337**	**1392**	27	?							
			**1283**	**1446**	81	?	**1427**	2853	23	None			
			955	1774	409	?							
			727	2002	637	15PG-THB							
			477	2252	887	18PG+H_2_O							

The MS signals in bold are the predominant fragments and those underlined as the next more abundant, other MS^2^ signals are more minor. NL = neutral loss. ?s denote undefined neutral losses that probably arise by cross ring fragmentations as noted previously [27]. (-THB) denotes the presence of a tetrahydroxybenzene unit in the neutral loss. * denotes that the NL differs by one amu from expected value.

## Supplementary Materials

The following are available online at https://www.mdpi.com/1660-3397/18/9/448/s1, Figure S1. Recovery of TPC after fractionation by Solid Phase Extraction and Sephadex LH-20; Figure S2. Co-chromatography of *m*/*z* [M − H]^−^ peaks at 745 and *m*/*z* [M + H]^+^ peaks at 1491 in SPE bound samples. Figure S3. Peak areas of even DP phlorotannin oligomers from DP10-24. Figure S4. Evidence for triple charged phlorotannin species.
