# Supplementary material for: Extraction, Enrichment, and LC-MSn-Based Characterization of Phlorotannins and Related Phenolics from the Brown Seaweed, Ascophyllum nodosum
Source: Mar Drugs. 2020 Aug 27;18(9):448. doi: 10.3390/md18090448 (PMC7551814; doi:10.3390/md18090448)
Supplement: Supplementary file 1 [file marinedrugs-18-00448-s001.pdf]

Supplementary Information

Article

Extraction, Enrichment, and LC-MS^n-Based Characterization of Phlorotannins and Related Phenolics from the Brown Seaweed, *Ascophyllum Nodosum*

J. William Allwood [1], Huw Evans [2], Ceri Austin [1] and Gordon J. McDougall [1,*]

[1] Plant Biochemistry and Food Quality Group, Environmental and Biochemical Sciences Department, The James Hutton Institute, DD2 5DA Dundee, UK; will.allwood@hutton.ac.uk (J.W.A.); Ceri.Austin@hutton.ac.uk (C.A.)

[2] Byotrol Ltd., Thornton Science Park, CH2 4NU Chester, UK; hevans@byotrol.com

* Correspondence: gordon.mcdougall@hutton.ac.uk; Tel.: +44-1382-568782

Received: 17 August 2020; Accepted: 24 August 2020; Published: date

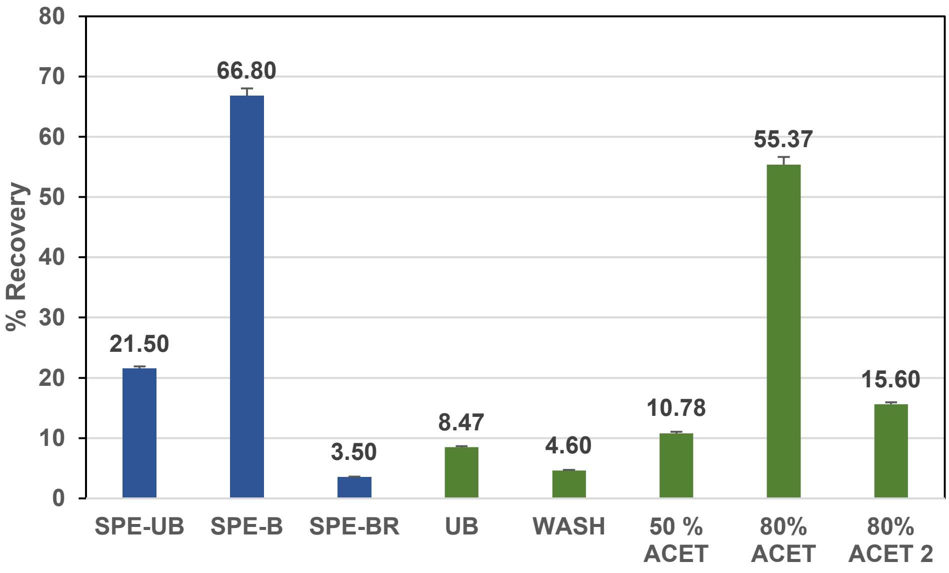

The SPE-Unbound sample includes the unbound and wash fractions.

Figure S1. Recovery of TPC after Fractionation by Solid Phase Extraction and Sephadex LH20

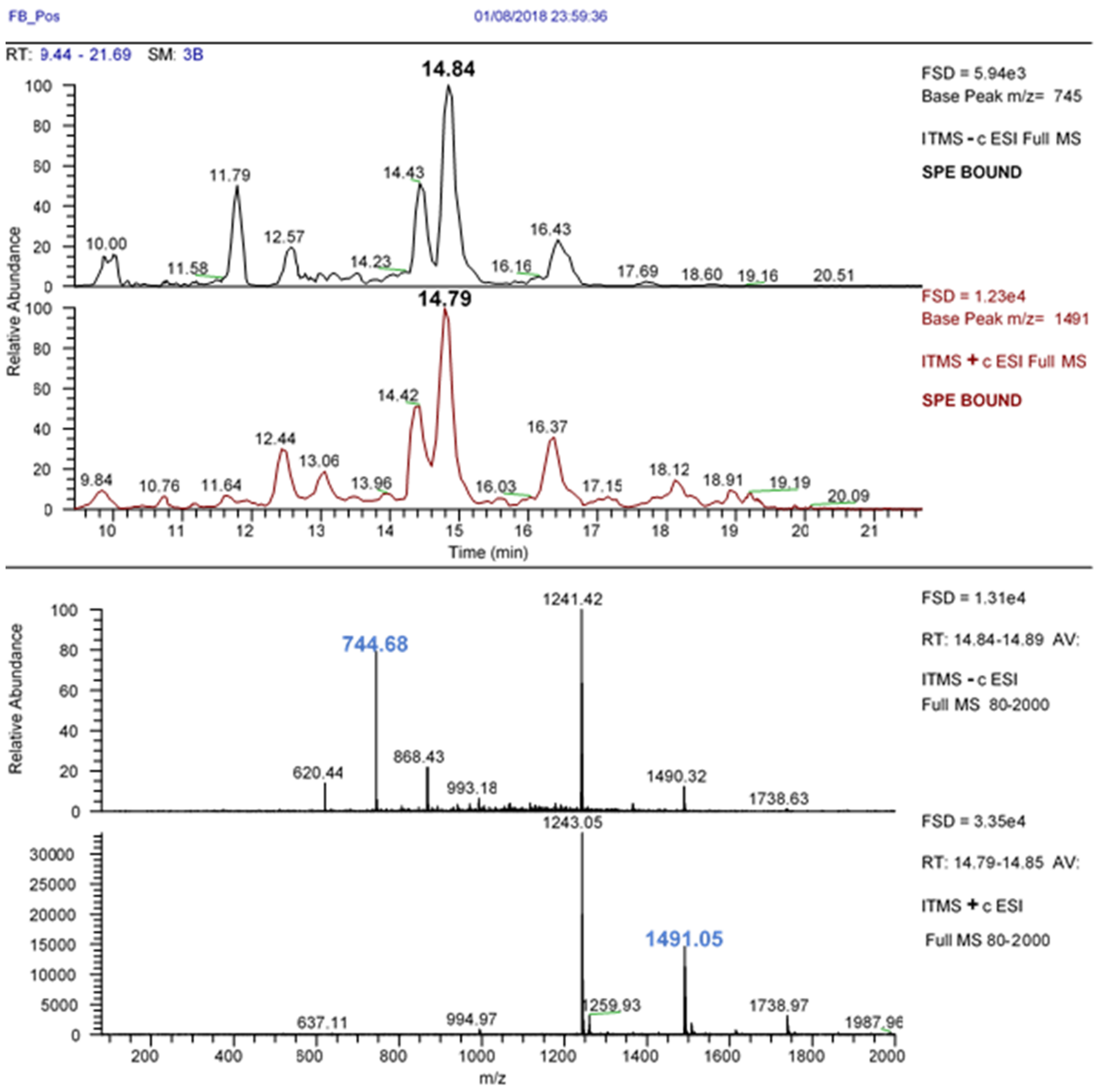

Figure S2. Co-chromatography of *m/z* [M-H]$^-$ peaks at 745 and *m/z* [M+H]$^+$ peaks at 1491 in SPE bound samples

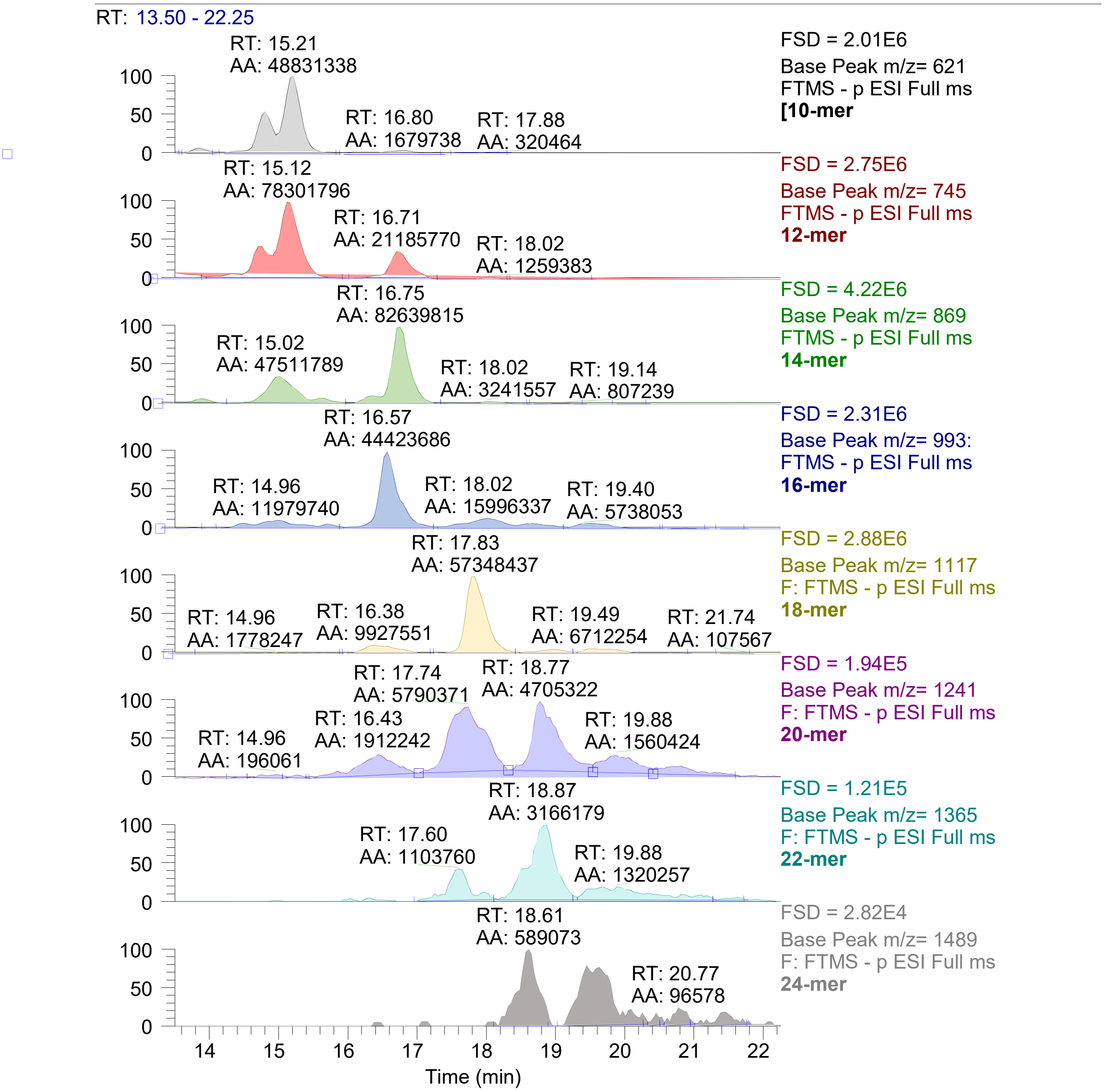

Dual peaks appear for most *m/z* species which suggests the presence of isomers of the different DP oligomers. For quantification, we summed the areas for these major putative isomer peaks but ignored other small peaks which may be due to in-source fragments within the MS spectra of other oligomers.

Figure S3. Peak areas of even DP phlorotannin oligomers from DP10 - 24

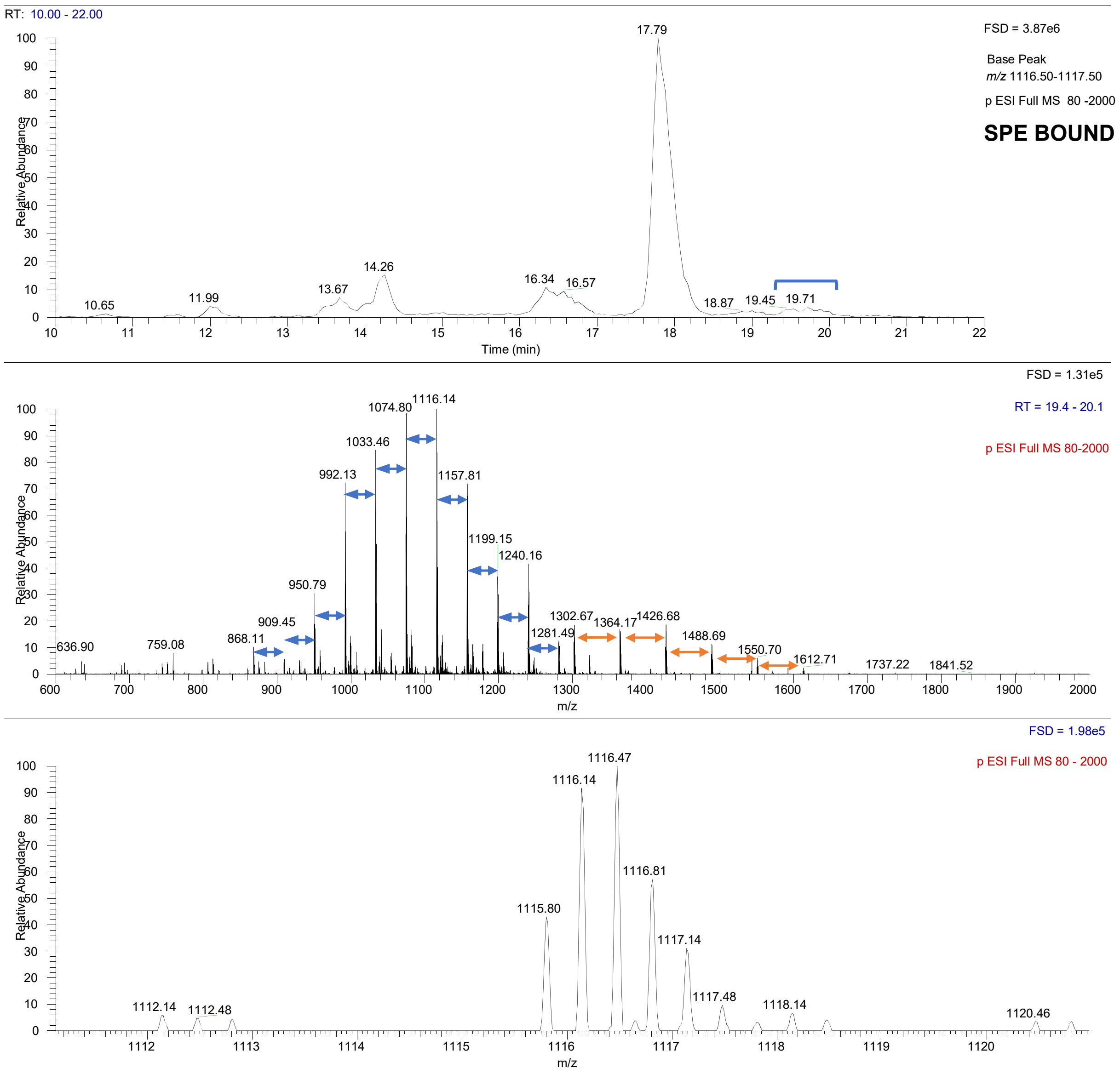

Blue arrows illustrate spacing of ~41 amu, orange arrows illustrate spacing of ~62 amu. Zoom scan in the bottom panel shows that the *m/z* signals are triply charged with 0.33 amu spacings.

Signals the series from *m/z* 868 up to 1281 showed separation of ~ 41 amu and were triply charged. The highest DP in this sequence was estimated at 31 and MW of 3846.

Signals from *m/z* 1302 upwards showed separation of 62 amu and were doubly charged. Therefore, the highest DP in this sequence was estimated at 26 and MW of 3226.

Figure S4. Evidence for triple charged phlorotannin species
